# Transcriptome sequencing reveals thousands of novel long non-coding RNAs in B cell lymphoma

**DOI:** 10.1186/s13073-015-0230-7

**Published:** 2015-11-01

**Authors:** Akanksha Verma, Yanwen Jiang, Wei Du, Lauren Fairchild, Ari Melnick, Olivier Elemento

**Affiliations:** Institute for Computational Biomedicine, Weill Cornell Medical College, 1305 York Avenue, New York, NY 10021 USA; Institute for Precision Medicine, Weill Cornell Medical College, 1300 York Avenue, New York, NY 10021 USA; Division of Hematology/Oncology, Department of Medicine, Weill Cornell Medical College, 1300 York Avenue, New York, NY 10021 USA; Department of Physiology and Biophysics, Weill Cornell Medical College, 1300 York Avenue, New York, NY 10021 USA

## Abstract

**Background:**

Gene profiling of diffuse large B cell lymphoma (DLBCL) has revealed broad gene expression deregulation compared to normal B cells. While many studies have interrogated well known and annotated genes in DLBCL, none have yet performed a systematic analysis to uncover novel unannotated long non-coding RNAs (lncRNA) in DLBCL. In this study we sought to uncover these lncRNAs by examining RNA-seq data from primary DLBCL tumors and performed supporting analysis to identify potential role of these lncRNAs in DLBCL.

**Methods:**

We performed a systematic analysis of novel lncRNAs from the poly-adenylated transcriptome of 116 primary DLBCL samples. RNA-seq data were processed using *de novo* transcript assembly pipeline to discover novel lncRNAs in DLBCL. Systematic functional, mutational, cross-species, and co-expression analyses using numerous bioinformatics tools and statistical analysis were performed to characterize these novel lncRNAs.

**Results:**

We identified 2,632 novel, multi-exonic lncRNAs expressed in more than one tumor, two-thirds of which are not expressed in normal B cells. Long read single molecule sequencing supports the splicing structure of many of these lncRNAs. More than one-third of novel lncRNAs are differentially expressed between the two major DLBCL subtypes, ABC and GCB. Novel lncRNAs are enriched at DLBCL super-enhancers, with a fraction of them conserved between human and dog lymphomas. We see transposable elements (TE) overlap in the exonic regions; particularly significant in the last exon of the novel lncRNAs suggest potential usage of cryptic TE polyadenylation signals. We identified highly co-expressed protein coding genes for at least 88 % of the novel lncRNAs. Functional enrichment analysis of co-expressed genes predicts a potential function for about half of novel lncRNAs. Finally, systematic structural analysis of candidate point mutations (SNVs) suggests that such mutations frequently stabilize lncRNA structures instead of destabilizing them.

**Conclusions:**

Discovery of these 2,632 novel lncRNAs in DLBCL significantly expands the lymphoma transcriptome and our analysis identifies potential roles of these lncRNAs in lymphomagenesis and/or tumor maintenance. For further studies, these novel lncRNAs also provide an abundant source of new targets for antisense oligonucleotide pharmacology, including shared targets between human and dog lymphomas.

**Electronic supplementary material:**

The online version of this article (doi:10.1186/s13073-015-0230-7) contains supplementary material, which is available to authorized users.

## Background

Gene expression profiling of diffuse large B cell lymphoma (DLBCL) has revealed broad gene expression deregulation compared to normal B cells. These studies identified two main DLBCL subtypes - activated B-cell like (ABC) and germinal center B-cell (GCB) – associated with distinct clinical outcomes [1]. They also identified involvement of other signatures, for example, a stromal signature [2]. These studies used microarray to measure gene expression and therefore only interrogated well-known and annotated genes. Additional recent studies used transcriptome sequencing (RNA-seq) to look for gene fusions and deregulated pathways in DLBCL [3].

In theory, RNA-seq data can be examined to look for novel, yet unannotated, transcripts. While it is generally thought that most of the proteins coding genes encoded in the human genome have been discovered, many yet unannotated long non-coding RNAs (lncRNAs) are thought to exist. LncRNAs are a type of ncRNA that are at least 200 bp long and are spread across the intergenic regions in the genome. Based on recent studies, some of these lncRNAs, despite being non-coding, are shown to play critical roles in disease specific epigenetic gene regulation, including in cancer biology [4]. For example, several lncRNAs interact with the Polycomb complex (PRC1/PRC2) to promote repression of gene expression [5, 6].

The discovery of novel lncRNAs is challenging for a number of reasons, including their frequent low expression, the algorithmic and statistical complexity of *de novo* discovery. Nonetheless, these challenges are being overcome and several groups have performed systematic analysis of lncRNAs in normal cells and also in primary tumors. A recent cross-cancer study by the Chinnaiyan group uncovered thousands of novel lncRNAs [7]. Another study by the Maher group identified a large number of novel lncRNAs in lung cancer [8].

In the present study, we sought to discover and characterize novel lncRNAs in DLBCL using a *de novo* transcript discovery strategy based on RNAseq of primary DLBCLs and normal B cell samples. We performed a systematic analysis of 116 tumors and used stringent filtering based on conventional characteristics of lncRNAs in terms of coding properties, length, exon counts, and expression levels to identify high-confidence novel lncRNAs. We identified a total of 2,632 novel DLBCL lncRNAs. Subsequently, we applied a broad range of analyses to these lncRNAs to further understand their function. For example, we looked for lncRNAs co-expressed with PRC2 components such as *EZH2* to identify potential PRC2 interaction partners. We analyzed the network of co-expressed protein coding genes to identify a potential function for each lncRNA. We inspected the local genomic neighborhood of lncRNAs to look for functional enrichment. We analyzed the correlation between lncRNAs and disease features such as tumor subtypes (ABC vs. GCB), DLBCL super-enhancers and how mutations in lncRNAs may impact their function. For the first time, a cross-species analysis of lncRNAs was performed and we found that a non-negligible fraction of human DLBCL lncRNAs is also expressed in canine lymphoma. We also identified novel features of lncRNAs, such as their significant overlap with transposable elements, especially within the last exon. Altogether, these analyses strongly suggest that novel DLBCL lncRNAs are functionally embedded within lymphoma gene networks and play important roles in lymphomagenesis and/or maintenance of the lymphoma phenotype. At a time where antisense oligonucleotides are starting to demonstrate clinical potential [9] this study provides a wealth of potential new anti-lymphoma pharmacological targets. The conservation of some of these lncRNAs in dogs suggests a potential route for developing anti-lymphoma strategies based on dog therapeutic trials.

## Methods

### Data

Computational analysis was performed on multiple sets of RNAseq data, including 116 TCGA dbGAP DLBCL tumor samples (dbGaP accession number phs000235.v6.p1 – dbGaP approval for this specific project was granted). The eight normal B-cell (four naïve B cells and four germinal center B cells) samples were obtained from a previous study dataset (GEO dataset: GSE45982) and 30 DLBCL cell lines (Additional file 1) were obtained from the Melnick lab. The naïve B cells (from tonsillar naïve B cells) and centroblast (from tonsillar germinal center B cells) were treated by magnetic bead cell separation and the total RNA was extracted for RNA-seq using Qiagen kits [33]. Polyadenylated RNA-seq was performed using the standard Illumina Truseq kits and samples were sequenced using HiSeq2000 with one to three samples per lane. RNA-seq data from dog lymphoma samples were obtained from DNANexus [27] and used for the cross-species analysis.

### RNA-seq and *de novo* transcript assembly pipeline

All RNA-seq analyses were performed using conventional RNA-seq analysis tools. All RNA-seq short reads were aligned to human reference genome (version hg19/GRCh37) using *STAR* [10]. Post alignment, the aligned reads were put through *de novo* transcript assembly and numerous bioinformatics tools, along with some in-house scripts for processing.

### *De novo* transcript assembly to obtain novel transcripts

The *ab initio* transcript assembly was performed using CuffLinks (v2.2.1) [8] in *de novo* mode to assemble transcripts for 116 DLBCL tumor samples and eight normal B cell samples. The assembled transcript fragments from the cohort of tumor and normal B cell samples, were merged to create a consensus transcriptome GTF file, using the tool *CuffMerge* [8]. This consensus transcriptome was parsed to filter out any previously annotated transcripts such as known protein coding genes and known lncRNAs. A consensus dataset of known annotations was created using protein coding gene annotations from UCSC, GencodeV17, RefSeq, and Ensembl, while known lncRNA annotations were obtained from Human lncRNA catalog-Broad institute. Using the tool *CuffCompare*, the assembled consensus transcriptome GTF was compared to the database of known annotations to obtain a consensus novel transcript GTF for further analysis.

### Filtering for novel lncRNA candidates

Stringent filtering was performed to control for artefactual and other background noise generated due to *de novo* assembly of the alignments, which may have been counted in as a novel transcript. Filtering was done for coding potential, transcript length, and number of exons. Coding Potential Assessment Tool (CPAT) [9] was used to compute the coding potential for each transcript. Given a FASTA input, CPAT uses logical regression model based on ORF size, Fickett score, and hexamer usage bias. Based on these, CPAT predicts each transcript’s coding property and assigns a coding potential score in the range of 0–1, with CPAT score <0.364 assigned for non-coding transcripts and >0.364 for protein-coding transcripts. The length of each non-coding transcript was also obtained from the results of CPAT, which was used in filtering for transcript length, selecting long sequences (> = 200 bp). An in-house script was used to count the number of exon per transcript from the assembled unannotated transcripts GTF and those with at least 2 exons or more were included for analysis. A final novel lncRNA GTF was then created with the filtered in transcript fragments. Using the novel lncRNA GTF as reference, novel lncRNA candidates were quantified for FPKM levels in all samples in DLBCL tumor, normal B cells, and DLBCL cell lines, using CuffLinks. Based on the obtained FPKM levels, each lncRNA expressed in at least two or more samples were established as the selected novel lncRNA candidates and used for all further analysis.

### Divergently transcribed lncRNAs

LncRNAs transcribed in an opposite orientation from the identified nearest protein-coding gene. These divergently transcribed lncRNAs were selected based on a two-step analysis. First, all lncRNAs whose first exons were within 2 kb of a protein-coding gene were selected. Then out of these, those which were transcribed in the antisense orientation from their nearest protein-coding gene were selected as divergently transcribed lncRNAs.

### Statistical analysis

Almost all of the statistical analysis for correlations, differential expression, and other statistical tests applied were performed using R statistical analysis software. Controls for false positives and significance stringency were applied accordingly based on each analysis. FPKM based differential expression analysis per lncRNA was performed on ABC and GCB classified samples using t test statistic. Multiple hypotheses testing correction was then performed on significantly differentially expressed lncRNA across ABC and GCB by adjusting for *P* values. Only transcripts differentially expressed across the subtypes with adjusted *P* value (FDR) <0.05 were considered.

### Repeat elements analysis

Transposable element reference used for comparison was first filtered to remove low complexity and satellite repeats, to focus on the major transposable elements sub families. BEDtools (v2.23.0 ) [11] intersect was used to obtain the exonic overlap between novel lncRNAs and transposable elements. Partial or complete exonic overlaps were only considered as valid overlaps. Another tool, RepeatMasker [12], was used to obtain sequence based overlap of TE with novel lncRNA for lncRNAs, giving the TE-derived percentage. Dividing the base-by-base TE overlap for a transcript by the total length of that transcript returned the TE-derived percentage. All transcripts, which showed some percentage of sequence based overlap with TE, were termed as TE-derived.

### Cross-species analysis

Dog transcriptome was reconstructed, using CuffLinks in *de novo* mode post alignment to CanFam3 genome build. This reconstruction was then filtered to obtain multiexonic transcripts using an in-house script. For a consistent comparative analysis, the tool LiftOver was used to convert genomic coordinates in BED format from human to canine, using the UCSC [13] chain file for hg19toCanFam3 as reference. Lifted over human lncRNAs were then intersected with the Dog transcripts using BEDtools intersect.

### Mutation analysis

SNVs in the exonic regions of the novel lncRNAs were identified using VarScan (minimal coverage of 8 and variant allele frequency of 0.2), based on the samtools generated pileup input format of the lncRNA regions. All SNVs present in the intronic regions were removed using BEDtools intersect. *SnpSift* from *snpEff* toolbox [14] was then used to remove the already annotated SNVs using the dbSNP annotations as reference, to obtain only novel mutations (SNVs). These novel SNVs were then used to create a mutated human genome reference using the GATK tool *FastaAlternateReferenceMaker* [15]. Based on the VCF with exonic mutations, this tool mutated the original reference, which was then used to create mutated lncRNA FASTA sequences. The program *gffread,* included in the CuffLinks package, was used to generate all FASTA sequences, given a GTF and corresponding genome reference.

Given the FASTA sequence for the native sequences of the lncRNAs and the mutated sequence of the lncRNAs, RNAfold was then used to compute the minimum free energy (MFE) of the secondary RNA structure in unit kcal/mol. A difference in the original/wildtype MFE and mutated MFE of each lncRNA transcript was then used to study the shift in the energies. Similar analysis was carried out for the exonic dbSNP mutations present in the lncRNAs, as a control for the study in the change of MFE.

### Visualization

Integrative Genomics Viewer (IGV browser v.2.3.34) [16] was used to visualize and document the genomic coordinates in various file formats.

## Results

### *De novo* transcript discovery identifies 2,632 novel lncRNA in DLBCL tumors

**Fig. 1:**
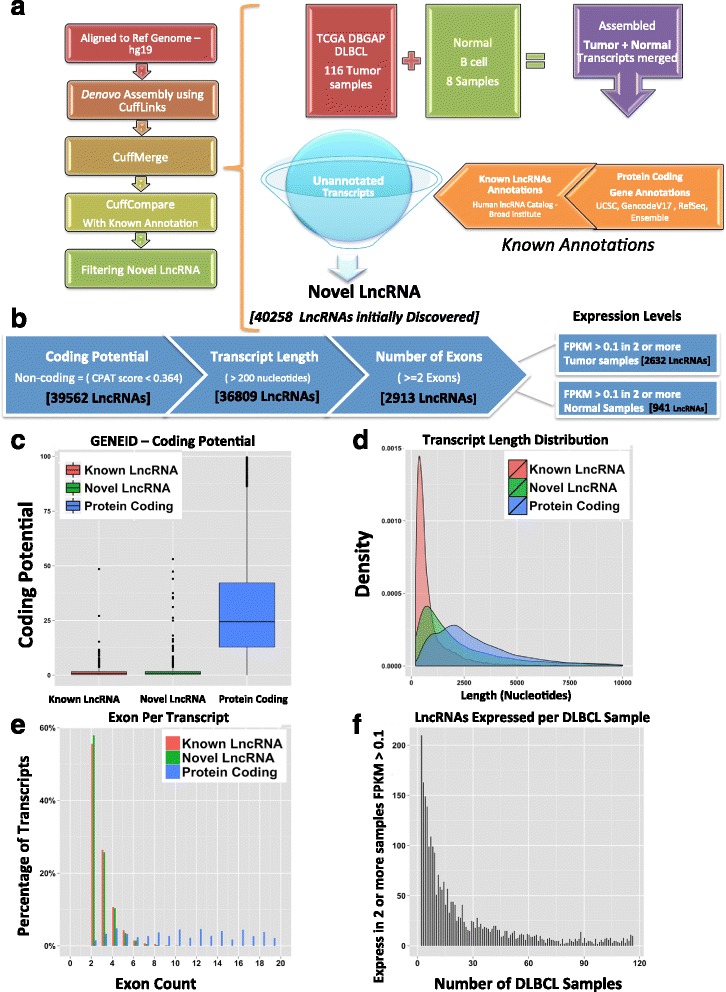
*De novo* transcript discovery identifies 2,632 novel lncRNA in DLBCL tumors. **a** A well-organized pipeline was used to discover and obtain candidate lncRNAs. Using aligned 116 tumor and eight normal B cell samples, the transcriptome was reassembled using CuffLinks in *de novo* mode. A cohort of the assembled transcripts for each sample was then created using CuffMerge and parsed out for novel unannotated transcripts using CuffCompare, given a consensus reference of all known annotations from various sources – UCSC, RefSeq, ensemble, and GencodeV17. Initially we discovered 40,258 unannotated novel transcripts. **b** Stringent filtering steps applied to the initially discovered novel transcripts to remove artefactual novel lncRNAs, based on selection of non-coding transcripts using CPAT, transcript length, and exon count. Post these, expression level filtering (FPKM > 0.1 in two samples or more) across each sample group was performed, respectively. **c** GENEID coding potential score was used to validate the coding potential comparing known LncRNAs, novel lncRNAs, and protein-coding genes. The known lncRNA emulate the novel LncRNAs’ low average coding potential score, while a higher average coding potential score for protein coding genes is observed. **d** Transcript length using a density plot was also compared – showing common patterns for known lncRNAs and novel lncRNAs, with protein coding gene, were much longer in length. **e** Based on the 2 exon on more cutoff, the bar plot shows all novel lncRNA, similar to known lncRNAs have at least 2 or more exons. Protein coding has a lot more exons compared to the known and novel lncRNAs. **f** Bar plot showing, selected lncRNA based on FPKM filtering (FPKM > 0.1 in two or more samples) in DLBCL tumor samples, ordered to show count of selected lncRNAs expressed per tumor sample (n = 116)

We hypothesized that *de novo* analysis of primary DLBCL RNAseq would help uncover novel lncRNAs. RNA-Seq reads from a cohort of 116 primary DLBCL tumor samples (dbGaP accession number phs000235.v6.p1) were aligned to human reference using an RNA-seq aligner (STAR) and were then subjected to *ab initio* transcript assembly [17]. We also processed eight normal B cell RNA-seq samples (four naïve B cells and four germinal center B cells) using the same analysis. The initial transcript sets were merged and only those transcripts that do not overlap any previously annotated protein-coding gene or known lncRNAs according to annotations from known gene databases (UCSC, GencodeV17, RefSeq, Ensembl, Human lncRNA catalog-Broad institute) were retained (Fig. 1a). This set of novel candidate lncRNAs was put through further stringent filtering based on established properties of a lncRNA, including coding potential, transcript length, and exon numbers, to obtain a specifically characterized group of lncRNAs (Fig. 1b). Novel candidate lncRNAs were first checked for coding potential to determine if they held coding properties, using CPAT [18]. LncRNAs which passed the CPAT coding potential score cutoff (<0.364) for non-coding genes, were selected for further analysis. Out of the non-coding transcripts, we selected transcripts with length of 200 bp or greater. Since it is possible that *de novo* assembly may have improperly assembled artefactual background noise, un-spliced pre-mRNA or gene extensions, only multiexonic transcripts were selected for further analysis. After applying these stringent filters, we were left with 2,913 novel DLBCL/normal B cell lncRNAs. We quantified the expression levels of these 2,913 lncRNAs in all tumors and all normal B cell samples and only retained lncRNAs that have expression (FPKM >0.1) in at least two or more samples in each of the sample groups. The FPKM threshold was chosen after analyzing the known lncRNAs, which show comparable levels of expression and other previously published lncRNA analysis in other cancers which also used FPKM cutoff of <0.1 [7, 8]. This analysis led to 2,632 lncRNAs expressed in tumors and 941 lncRNAs expressed in normal B cell samples (Fig. 1b). All further analyses below were performed on the 2,632 tumor-expressed lncRNAs (Additional file 2).

Coding potential scores for the 2,632 novel lncRNAs, known lncRNAs, and protein-coding genes were generated using GENEID [19] for cross-validation. This showed novel lncRNAs to emulate the low average coding potential score of the known lncRNAs in comparison to the higher average coding potential score characterizing the protein coding genes (Fig. 1c). We also verified that our novel lncRNAs follow similar length density distribution of known lncRNAs (even though they tend to be longer) and are on average shorter than protein-coding genes (t-test, *P* <2.2e-16) (Fig. 1d). Finally, we compared the number of exons of our lncRNAs with the numbers for known lncRNAs and protein-coding genes: the novel lncRNAs emulate known lncRNAs, with the majority of them between 2 and 4 exons, while protein-coding genes have a much larger exon count (Fig. 1e). When we counted how many lncRNAs are expressed in each tumor, we found that many lncRNAs were expressed in a few tumors while a smaller number were expressed across a large number of tumors (Fig. 1f). When we compared our 2,632 novel lncRNAs with those uncovered by Iyer *et al.* [7] in 27 tissues and cancer types but not in B cell lymphomas, we only found 17 % overlap suggesting that the vast majority of our DLBCL novel lncRNAs are DLBCL specific (Additional file 3: Figure S1).

### Many of the novel DLBCL lncRNAs are tumor-specific

**Fig. 2:**
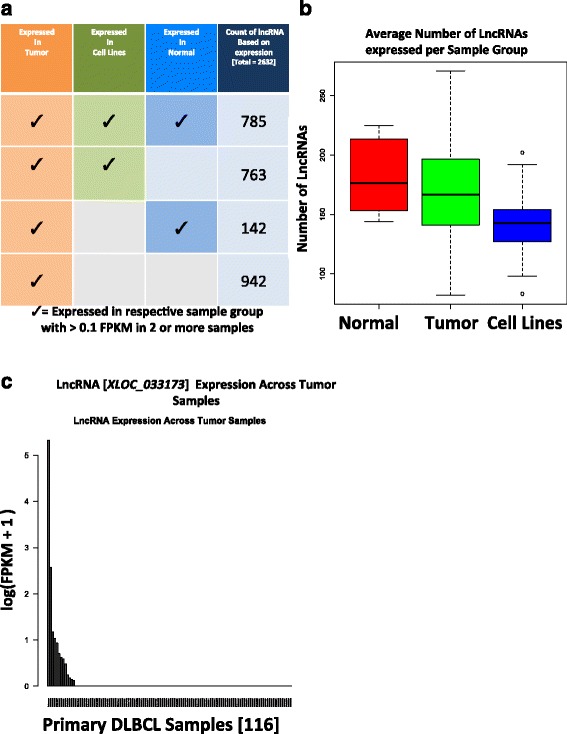
Many of the novel DLBCL lncRNAs are tumor-specific. **a** Selected novel lncRNA candidates were quantified for FPKM levels across each sample group. Filtering based on FPKM cutoff of FPKM >0.1 in at least two samples, in each sample group, respectively, showed 785 lncRNAs commonly expressed in tumors, normal, and cell lines. Also 763 lncRNA expressed in tumors and cell lines, while 142 lncRNAs expressed in normal and tumor, with 942 of the lncRNAs expressed uniquely only in tumors. **b** Number of lncRNAs expressed per sample with each sample group, plotted to show the expression across each sample group – with normal B cell sample group with higher average number of lncRNA expressed per sample, followed by tumor sample group and then cell lines. **c** An example of lncRNAs with exceptionally high expression (> = 10 standard deviation above mean) in specific tumor samples (out of 45 such lncRNAs) across all tumor samples

We further sought to subdivide the 2,632 tumor-expressed lncRNAs based on their expression in normal B cells and DLBCL cell lines. In the latter case, we quantified expression levels of the 2,632 lncRNAs in a panel of 30 DLBCL cell lines. As before, a lncRNA was considered expressed if at least two samples within the cell line group had expression >0.1. Altogether, we found that 763 lncRNAs are expressed in tumors and cell lines but not in normal B cells. Across the normal B cell subtypes – we found that 718 lncRNAs of the 2,632 novel lncRNAs are expressed in centroblasts (FPKM >0.1 in two or more samples) and 575 in naïve B cells. We also found that only 927 lncRNAs were expressed both in tumors and normal cells. Out of these 927 lncRNAs, 334 were significantly differentially expressed across the two normal subtypes – naïve B Cells and GCB (FDR <0.05) and clustering (supervised clustering, using hclust function; method ward) based on the lncRNAs recapitulated the respective sample groups (Additional file 4: Figure S2). Another 942 lncRNAs are uniquely expressed in DLBCL tumors, that is, not in normal B cells or cell lines (Fig. 2a). Finally, 785 lncRNAs were expressed in tumors, cell lines, and normal B cells and the remaining was expressed in DLBCL tumors and normal B cells but not in cell lines. Comparing all 2,632 tumor-expressed lncRNAs against normal B cells (as a single group) showed 1,090 lncRNAs significantly differentially expressed (FDR <0.05) and clustered across the sample groups (supervised clustering, using hclust function in R; method ward), indicating 41 % of these differentially expressed lncRNAs across normal and tumors may indeed contribute to lymphomagenesis (Additional file 5: Figure S3).

We analyzed the 785 ubiquitously expressed lncRNAs and asked how many of them were expressed in each sample. We found normal samples to have the highest on average number of expressed lncRNAs per sample, then tumor samples followed by cells lines which have the least, respectively (Fig. 2b). This result may suggest that many lncRNAs are transcriptionally silenced in rapidly proliferating cells. Alternatively, this result may reflect differences in cell type complexity and transcriptional heterogeneity between these cell types.

We nonetheless found 45 specific lncRNA that show exceptionally high expression (> = 10 standard deviation above mean 2.17 FPKM) in a subset of tumor samples. Expression levels of one such lncRNA, *XLOC_033173*, is shown in Fig. 2c. There were 45 lncRNAs (Additional file 2) with such pattern of expression across tumor samples. We speculate that some of these lncRNAs may be involved in structural alterations that led to their over-expression [2] and that some of these outlier lncRNAs may drive these tumors. Indeed, when we overlapped these 45 lncRNAs with published data from genome wide copy number analysis performed in primary DLBCL tumors [5], using array CGH, we found 33 out of the 45 lncRNAs overlapped with known recurrently amplified regions in DLBCL.

**Fig. 3:**
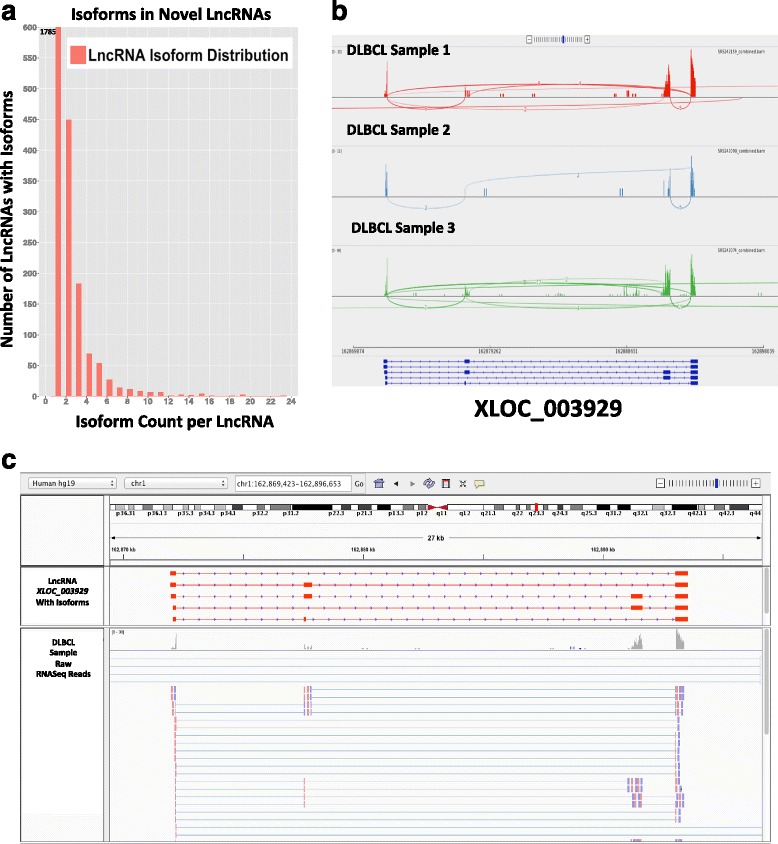
Quantifying present isoforms for the novel lncRNAs. **a** With most lncRNAs being single transcripts, about 35 % of the novel lncRNAs seemed have isoforms. (Plot axis cutoff, number shows lncRNAs with at least 1 isoform). **b** Sashimi plot for one of the novel lncRNAs (XLOC_003929) shows expression and clearly defined slice sites for a novel lncRNA with five isoforms, across three tumor samples. **c** An IGV browser screenshot shows the RNA-seq reads aligned to the same novel lncRNA (XLOC_003929) from (b), confirming expression patterns along the splice-site, as per the detected isoforms

Finally, we observed that the 2,632 unique novel lncRNAs were in fact derived from 4,608 distinct transcripts, indicating presence of multiple isoforms for some of these lncRNAs. While most of our lncRNAs have a single isoform, many lncRNAs had more than one isoform, with the maximum of 23 isoforms for one of our lncRNAs (Fig. 3a). For example, visualizing some of these isoforms using sashimi plot (Fig. 3b) and raw reads (Fig. 3c) for lncRNAs *XLOC_003929* across multiple tumor samples, we observed expression patterns across clearly defined spliced junctions for various isoforms. This suggests that like protein-coding genes, lncRNAs alternative splicing is used to increase transcriptional (and perhaps functional) complexity.

### Integrative analysis reveals potential functions of DLBCL specific novel lncRNAs

**Fig. 4:**
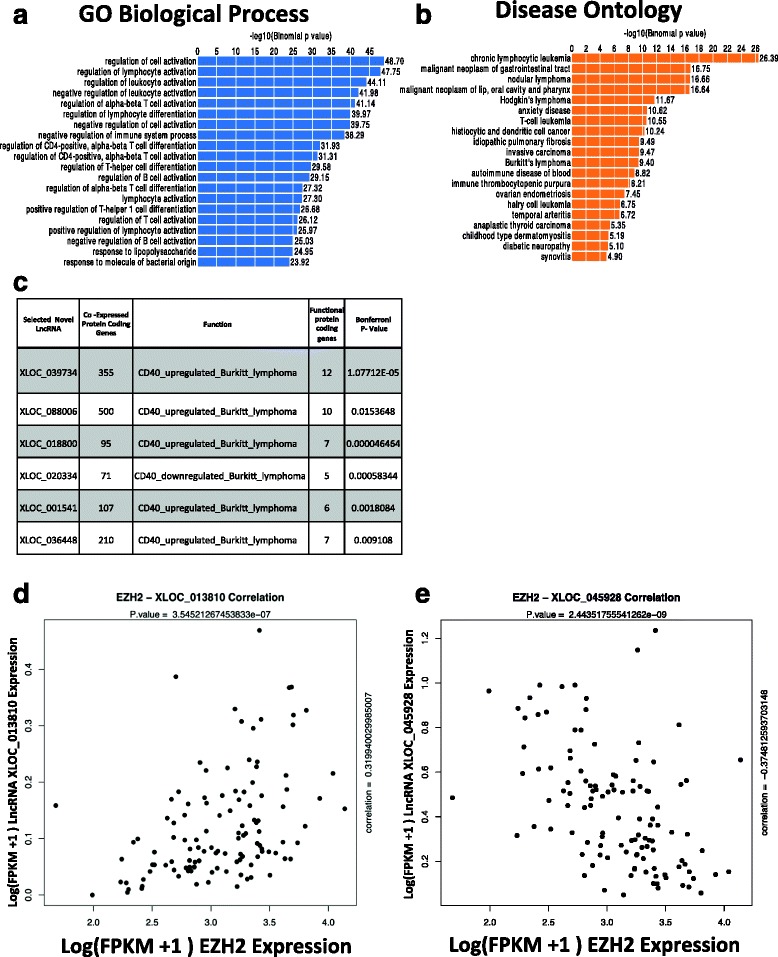
Integrative analysis reveals potential functions of DLBCL specific novel LncRNAs. **a** Biological processes GO ontology from GREAT, shows enrichment for various B cell and lymphoma-related pathways based on the nearby genes possibly regulated by the novel lncRNA. **b** Disease ontology from GREAT also shows lymphoma-specific disease pathways enriched based on gene in proximity to the novel lncRNAs. **c** Example of some lncRNAs with significant *P* values (Boniferroni, *P* value <0.05) which possibly regulate the functional pathways related to CD40- shown here with number of co-expressed protein coding genes for each, the related CD40-functional pathway, the subset of those co-expressed genes present in the pathway gene set. **d** Example of a significantly positively correlated lncRNA expression with *EZH2* expression across DLBCL tumor samples. **e** Example of significantly negatively correlated lncRNA expression with *EZH2* expression across DLBCL tumor samples

We then reasoned that analyzing our novel lncRNAs in the context of the genomic and co-expression/network could help potentially uncover their functions. For example, we noticed that a small but significant fraction of our lncRNAs (n = 166) are transcribed divergently (see Methods) from nearby protein coding genes, such as *RELA* (Additional file 6: Figure S4), perhaps contributing to regulating their gene expression. To expand on this analysis, we first examined the function of genes located nearby novel lncRNAs in the genome by using the computational tool GREAT [20]. GREAT analysis using Gene Ontology revealed gene functions in the vicinity of novel lncRNAs were related to immune cell activation and differentiation (Fig. 4a). Analysis using a disease-oriented ontology revealed that novel lncRNA neighborhoods are enriched with lymphoma or other immune cell malignancy genes (Fig. 4b). This suggests that novel DLBCL lncRNAs are not randomly located in the genome but preferentially located near genes with key functions in B cells and malignant B cells, perhaps contributing to the regulation of the function and expression of these genes.

To further explore the potential function of novel lncRNAs we performed a systematic co-expression analysis with known protein-coding genes. Correlating each lncRNA expression to known protein coding gene expression generated sets of significantly co-expressed (Spearman correlation; FDR <0.2) genes for each novel lncRNA (Additional file 7). About 88 % of the lncRNAs showed significant correlation with at least one protein-coding gene. We performed pathway analysis on the set of co-expressed protein coding genes for each lncRNA using Gene Ontology and lymphoid biology gene sets from the Staudt lab [21]. Overall, 43 % of the lncRNAs with co-expressed protein coding genes showed enrichment for at least one or more specific functional pathways (Additional file 8) such as CD40 upregulation and CD40 downregulation pathways (Fig. 4c). Reasoning that the function may in theory be transferred between co-expressed protein coding genes and lncRNAs, this means that a bit under half of our novel lncRNAs can be given at least one putative function. We then specifically examined correlations between our lncRNAs and expression of *EZH2*, a transcriptional repressor, implicated in tumorgenesis in DLBCL due to frequent mutations and with known interactions with lncRNAs [22, 23]. Since co-expressed genes are known to be enriched for physically interacting gene products, we reasoned that positive correlations might uncover potential *EZH2* interaction partners [24, 25] or alternatively lncRNAs regulated by the same transcriptional mechanisms as EZH2. Negative correlation might represent *EZH2* repressed lncRNAs. We identified 682 lncRNAs to be significantly correlated (FDR <0.2) with *EZH2*, out of which 251 positively (example shown in Fig. 4d) correlated and 431 negatively correlated (example shown in Fig. 4e) [26]. In a siRNA knockdown analysis of EZH2 in four DLBCL cell lines - OCI-Ly7, Farage, SUDHL5, and WSUDLCL2, 182 of the lncRNAs negatively correlated with EZH2 were seen as upregulated (log2 fold change siEZH2/ control >1). This number was significantly higher than expected by chance according to the hypergeometric test (*P* <0.011), supporting the hypothesis that many lncRNAs negatively co-expressed with EZH2 may indeed be repressed by EZH2. Similar analysis with *BCL6* expression across tumors resulted in 323 negatively correlated lncRNAs (FDR <0.2). In a siRNA knockdown analysis of *BCL6* in OCI-LY1 DLBCL cells [27], 104 of these lncRNAs were also seen to be upregulated (log2 fold change siBCL6/siNT >1) at 24 h time point *BCL6* knockdown, while 48 of them are upregulated (log2 fold change siBCL6/siNT >1) at a 48 h time point knockdown of *BCL6*. As with EZH2, these numbers were significantly higher than expected by chance according to the hypergeometric test at 24 h (*P* <0.031) and 48 h time points (*P* <0.032). These negatively correlated lncRNAs are potential novel *BCL6* targets since *BCL6* is an obligate repressor [16].

### Novel DLBCL lncRNAs are frequently DLBCL subtype specific and enriched at DLBCL super enhancers

**Fig. 5:**
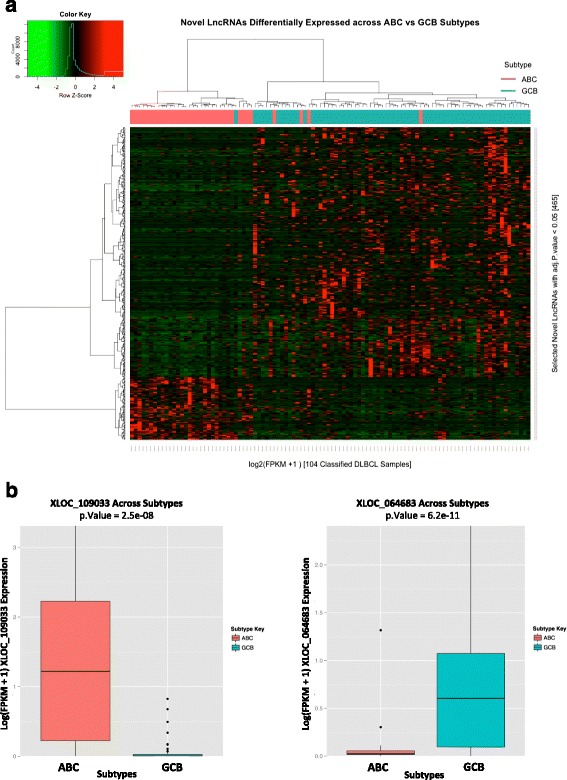
Novel DLBCL lncRNAs are frequently DLBCL subtype specific. **a** Heatmap showing overall differential expression for the significantly differentially expressed lncRNAs (n = 465) across 104 ABC and GCB classified samples, respectively. **b** Comparing novel lncRNA with significantly (adjusted *P* value <0.05) higher mean expression in ABC subtype classified tumor samples (n = 35 out of 104 classified samples) **c** Novel LncRNA with significantly (adjusted *P* value <0.05) higher mean expression in GCB subtype classified tumor samples (n = 69 out of 104 classified samples)

In DLBCL, differentiation block of B cells at different stages at least partially characterizes its known subtypes - ABC and GCB [1]. Both subtypes are known to have distinct prognosis, as a result of known variations in their gene profiles and association with distinct signaling pathways. We first classified 104 tumor samples (Additional file 9) into either GCB or ABC based on the published ABC/GCB classic expression based signature [28]. Using supervised analysis, we sought to discover novel ABC- or GCB-specific lncRNAs and identified 465 such lncRNAs (FDR <0.05; see Methods; 1,934 lncRNAs were obtained using FDR <0.2). Heatmap plots with unsupervised clustering (using hclust function in R) of the primary tumor samples, confirmed the pattern of subtype specific gene expression for these 465 significantly differentially expressed lncRNAs across ABC and GCB (Fig. 5a). Figure 5b illustrates examples of GCB and ABC-specific lncRNAs. This analysis confirms that many of our lncRNAs are not random and behave similarly to protein coding genes. Additionally, a similar analysis performed using 7,806 out of the 15,851 known lncRNAs (Broad institute Human Catalog, GENCODE V17) expressed >0.1 FPKM in at least two or more tumor samples, identified subtype specific known lncRNAs, with 891 (FDR <0.05; 2,088 lncRNAs were obtained using FDR <0.2) significantly differentially expressed and clustering (unsupervised clustering using hclust method in R; method ward) across the two subtypes (Additional file 10: Figure S5).

We also analyzed the correlation between novel lncRNAs and 283 DLBCL super-enhancers [29]. We found that 81 super-enhancers overlapped with our novel lncRNAs. Upon shuffling the enhancer locations 1,000 times we found that only 17 shuffled super enhancers on average overlap with lncRNAs (*P* <0.001). We conclude that DLBCL super-enhancers are enriched in novel lncRNAs, as illustrated in the *BCL6* upstream region (Additional file 11: Figure S6). We speculate that the open chromatin at these super-enhancers perhaps combined with cryptic promoters may facilitate lncRNA expression. We note that lncRNAs as defined here are different from shorter non-polyadenylated and non-spliced enhancer RNAs (eRNA) [30].

### Novel lncRNAs overlap with key histone marks, transcriptional regulators, and independently derived transcripts

To provide further support for the existence and functional role of our novel lncRNAs, we examined whether these the genomic loci of these novel lncRNAs were enriched of specific histone marks or bound by certain transcription factors.

**Fig. 6:**
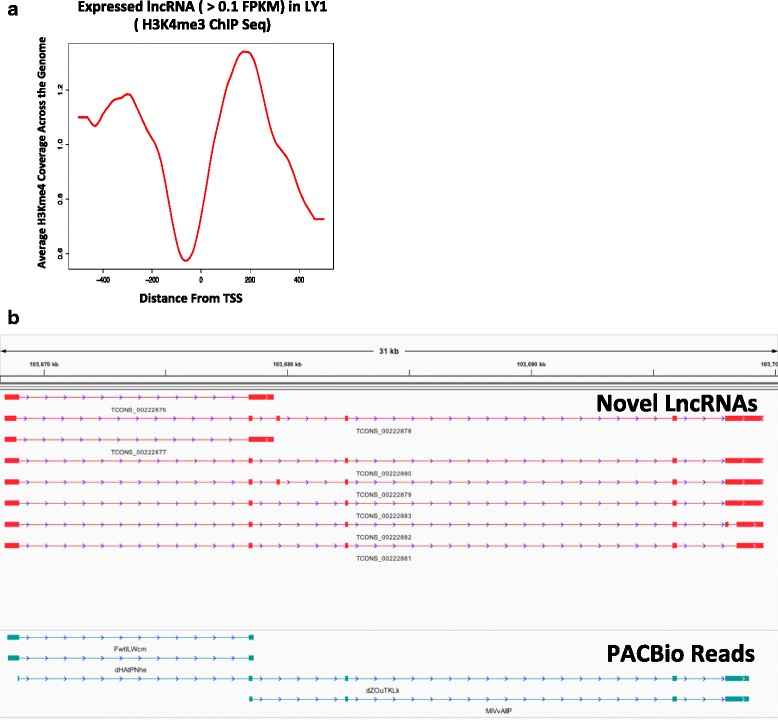
Novel lncRNAs overlap with key histone marks, transcriptional regulators, and independently derived transcripts. **a** Transcription Start Site plot showing average H3K4me3 read coverage across the entire genome at and around the TSS of the novel lncRNAs, with a nucleosome-free region slightly upstream of the TSS and a +1 nucleosome downstream. **b** IGV screenshot showing a well conserved overlapping lncRNA in the independent PacBio-derived transcript from (**c**)

H3K4me3 ChIP-seq data in DLBCL cell line OCI-LY1 (publicly available GEO data: GSE29282) [18] together with the 2,632 lncRNAs were used to further validate our lncRNAs. We created a transcription start site (TSS) plot (Fig. 6a) that reflects average H3K4me3 read coverage across the genome at and around the TSS of novel lncRNAs. Such plots, when determined from well-annotated protein coding genes (hg19 RefSeq) (Additional file 12: Figure S7), show a nucleosome-free region slightly upstream of the TSS and +1 nucleosome downstream. The TSS plot at the novel lncRNAs show a similar pattern, thus supporting the inferred TSS location for our novel lncRNAs and their validity as novel genes.

We also analyzed known lymphoma oncogenes - NFkB [31] and STAT3 [32] - to find out if their expression could be regulated by these oncogenes. Since we do not have NFkB binding data in lymphoma cells, we used NFkB binding data in lymphoblastoid cells from ENCODE [33] instead. We found that the overlap between our novel lncRNAs and NFkB ChIP-seq peaks (6,959 peaks) was limited but nonetheless existent (7 % peaks). For STAT3 ChIP-Seq peaks (6,256 peaks) in DLBCL cell line OCI-LY7 (publicly available GEO data: GSE50723) [20], we also found a small fraction of overlap (3 % out of the 2632 lncRNAs) with the novel lncRNAs.

To further validate our novel lncRNAs, we looked for overlap between them and long read (PacBio)-derived transcripts from lymphoblastoid cells [34]. We found that 10 % of our novel lncRNAs overlapped with the PacBio data. Most importantly, visualization showed clear conservation of the overlapping novel lncRNA structures in these independently derived samples (Fig. 6b).

### Role of transposable elements in lncRNAs

**Fig. 7:**
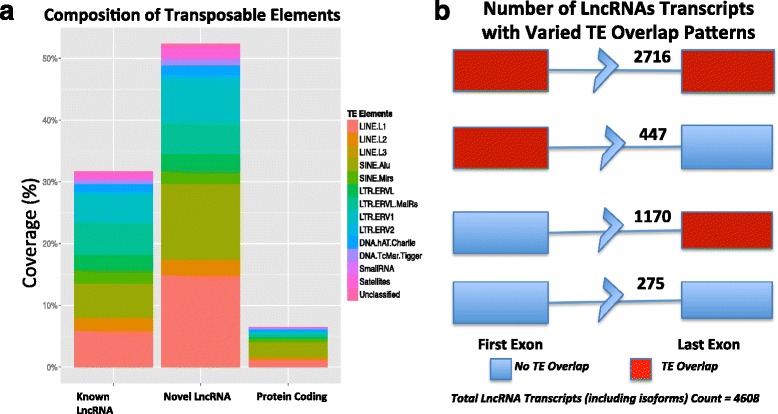
Role of TEs in lncRNAs. **a** Sequence-based TE content was calculated using RepeatMasker in known lncRNAs, novel lncRNAs, and protein-coding genes. Novel lncRNAs (53 % TE content), similar to known lncRNAs (30 % TE content) showed higher TE content compared to protein coding genes (6 % TE content). **b** Varied novel lncRNA exons and TE overlap patterns were noted, with a higher chance of a last exon overlap with a TE

Repetitive DNA sequences, also known as transposable elements (TE) are abundantly found in the genome. It has been reported by others that several lncRNAs contain transposable elements, for example, PCAT1 [35, 36]. In some cases, it is thought that TE may mediate the function of lncRNAs, allowing them to recognize and bind to target mRNAs containing TEs [37]. When we examined the exonic overlap between our lncRNAs and TEs using BEDtools [38] intersect, we found that up to 93 % of the lncRNAs overlap with TEs by 1 bp or more. Increasing the overlap threshold to 10 bp we found similar TE overlap of approximately 90 %. Overall, nearly half (53 %) of the lncRNA transcript sequences are TE-derived, that is 53 % of lncRNA nucleotides originate from TEs (Fig. 7a; see Methods). This fraction is much lower with protein-coding genes but more comparable to known lncRNAs (Fig. 7a). We further investigated the nature of the overlap and found that the last exon of novel lncRNAs vastly more frequently overlaps with TEs than the first exon (*P* <2.2e-16, chi-square test; Fig. 7b). Overall, these results hold for known lncRNAs albeit to a lower extent. We find that approximately 82 % of known lncRNAs (from Broad institute Human Catalog, GENCODE V17) overlap with TEs (using BEDtools intersect) and approximately 30 % of whose sequence is derived from TEs (using RepeatMasker; Fig. 7a). Likewise we observe that in known lncRNAs, the last exon overlaps more frequently with TEs than the first exon (*P* <2.2e-16; chi-square test) (Additional file 13: Figure S8), even if overall first and last exon overlap with TEs is lower in known lncRNAs compared to our novel lncRNAs. Last exon overlap with TEs is a previously unappreciated feature of lncRNAs and is compatible with lncRNA using cryptic polyadenylation signals [38] contained within TEs [39] and may in fact contribute to the biogenesis of novel lncRNAs (including disease-specific ones) on a scale that was not yet appreciated.

### Cross-species identifies conserved lncRNA transcription between dog and human lymphomas

**Fig. 8:**
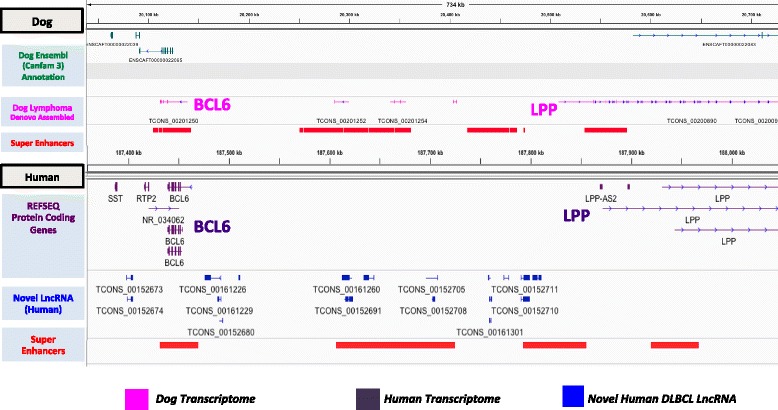
Cross-species identifies conserved lncRNA transcription between dog and human lymphomas. **a** Ensembl Dog (Canfam3) GTF show the homologs of *BCL6* and *LPP* genes, reconstructed dog lymphoma transcriptome, shows traces of human lncRNA overlap with known gene annotations such as *BCL6* and *LPP* gene as reference, transcripts in between the two show overlap with some of the lifted over human lncRNAs in Dog genome. About 9 % the human lncRNAs were identified with some overlap in the dog lymphoma transcriptome

With the aim to further affirm the existence of these DLBCL specific novel lncRNAs, we performed a cross-species analysis with canine lymphoma samples to assess conservation of these lncRNA across the same tumor type in both species. We analyzed dog lymphoma RNA-seq samples [40] because dogs get lymphomas whose features are similar to human lymphomas [41]. We reasoned that identifying conserved features between human and dog lymphoma lncRNAs may help underscore the importance of these features. Thirteen dog lymphoma samples were aligned to the dog genome (CanFam3), put through *de novo* transcript assembly and filtered to obtain multiexonic transcripts, similar to the novel lncRNA pipeline we used for human DLBCL analysis. For a uniform comparison across human and dog genomes, the Liftover tool was used to convert human novel lncRNAs to the dog genome coordinates and BEDTools was used to assess overlap. We found that 244 (9 %) human novel lncRNAs lifted over to CanFam3 showed at least partial exonic overlap with dog transcripts, where not the entire transcript overlaps but few exons or parts of exons overlap (using BEDTools intersect with specific bp overlap cutoff of 10 bp). Interestingly, as exemplified in the case of lncRNAs near the *BCL6* oncogene (Fig. 8), dog lncRNAs and human lncRNAs tend to be found in the same regions but are not located at the same exact location and do not necessarily overlap in terms of structure. That we nonetheless found 244 lncRNAs with at least one overlapping, sequence-similar exon is important since it is possible that novel DLBCL-specific lncRNA may represent interest targets for antisense pharmacology [9 ] and may enable cross-species clinical trials of such antisense oligonucleotides.

### Mutation analysis suggests that mutations in lncRNAs stabilize their structure

**Fig. 9:**
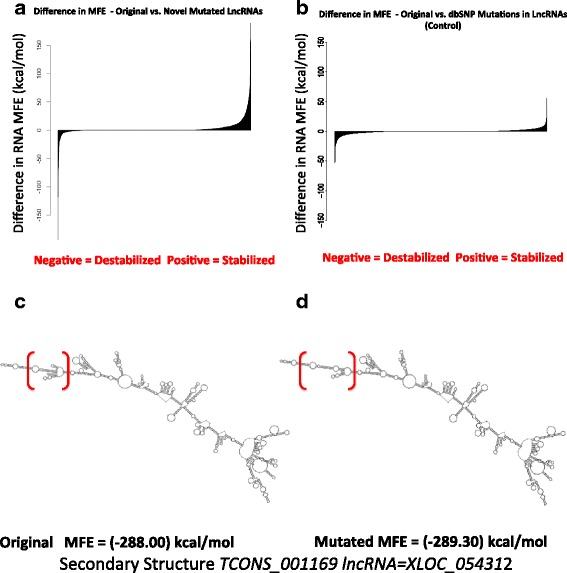
Mutation analysis suggests that mutations in lncRNAs stabilize their structure. **a** Difference in MFE (in kcal/mol unit) of native novel lncRNA sequences and mutated sequence with novel mutations shows a shift towards positive end of the scale, indicating stabilization of the secondary RNA structure of the lncRNAs by the novel mutations. **b** As a control, difference in MFE (in kcal/mol unit) of native novel lncRNA sequences and known dbSNP annotated mutations show a shift towards the negative end of the scale, indicating destabilization of the secondary structure by the dbSNP mutations. **c** Secondary structure of the original sequence of a lncRNA using RNAfold. **d** Secondary structure of the mutated sequence for lncRNA shown in (c). The slight change in MFE due to the mutations is also projected in the MFE secondary RNA structure, as highlighted

We wondered whether mutation analysis of lncRNAs would reveal lncRNA mutations and whether it would be possible to understand what these mutations may do functionally. Using VarScan [42] with default parameters, we performed a systematic mutation analysis of our lncRNAs and found a total of 9,713 novel candidate mutations within exonic regions of these lncRNAs after filtering out all known polymorphisms from dbSNP (release 142). Upon further filtering of variants found in our eight normal B cell RNA-seq samples (analyzed with the same parameters), we were left with 9,447 tumor-specific candidate mutations. This analysis suggests that lncRNAs may harbor frequent mutations (SNV) in DLBCL. We note, however, that this analysis is limited by the absence of matched normal comparators for our RNA-seq cases and that it cannot be excluded that some of these SNVs are in fact rare germline SNPs or sequencing errors. Out of these novel 9,447 SNVs, about 60 % of them re-occurred in at least 10 % of the tumor samples. A hotspot analysis, to find mutation frequency over a window of 100 bp, revealed 1,805 mutation hotspots with at least six or more mutations (either from independent samples or from the same sample). This suggests significant clustering of mutations in novel lncRNAs. We then wondered whether putative mutations in these lncRNAs would stabilize or destabilize the lncRNA secondary structure, leading to dysregulation of possible target genes and pathways. A Minimum Free Energy Score (MFE) was calculated for the native lncRNA sequence and the corresponding lncRNA sequence with one or more novel mutations using the tool RNAfold [43] and difference in MFE was calculated for each lncRNA. While many mutations did not seem to show any change in the MFE post mutation, a shift was evident toward stabilization of secondary RNA structures due to the positive difference in the MFEs of the original lncRNA and the mutated lncRNA (Fig. 9a). As a control, we performed the same analysis using 9,447 randomly selected dbSNP variants (common polymorphisms) detected in our samples in these lncRNAs and found that indeed the dbSNP variants were less likely to stabilize lncRNAs than the novel variants (*P* <2.2e-16; Wilcoxon test; Fig. 9b). Altogether these results identify a yet unappreciated potential role for DLBCL mutations in stabilizing lncRNAs, perhaps helping epigenetic mechanisms such as those mediated by *EZH2* in promoting lymphomagenesis and maintaining the tumor identity [44]. Figure 9c and d illustrates how slight stabilizing changes in the MFE of a novel lncRNA’s structure due to the novel mutations correlate with structural changes between the original and the mutated lncRNAs.

## Discussion

In this study, we identified 2,632 novel, multi-exonic candidate lncRNAs expressed in more than one DLBCL tumor. We found that a majority of our novel DLBCL specific lncRNAs seemed to have significant correlations with known data sets (Additional file 14), which suggests many of these discovered novel lncRNA potentially hold regulatory functions in DLBCL. H3K4me3 density (obtained by ChIPseq in a DLBCL cell line) at and around the predicted TSS of these lncRNAs shows a similar pattern compared to known protein coding genes, indicating their potential roles as active genes. These analyses provide a strong validation for the potential role and activity of these lncRNAs in DLBCL tumorgenesis and possibly tumor maintenance. Gene correlations with protein-coding genes show a large fraction (>80 %) of lncRNAs to be significantly co-expressed with at least one gene, suggesting potential co-regulation of genes not only nearby but also in the distant genome and their strategic positioning along these potent co-regulated genes. A significant number (43 %) of our lncRNAs show enrichment for important DLBCL-related functional pathways based on the co-expressed protein coding genes. Studying some of these pathways should provide insight on the specific functions of these lncRNAs and their roles. Some of these lncRNAs also expressed in cell lines, make them tractable targets to be studied in future experimental testing, for example to identify their tumor driver role or their regulatory functions.

In accordance with previous studies, our findings show lncRNAs to have a significant amount of overlap with transposable elements, which we hypothesize, may help define the regulatory functions for some of these novel lncRNAs. The preferential last exon overlap may also suggest a role for repeat element in birth of new genes, perhaps as a result of polyadenylation signals contained in these repeat sequences.

Finally, while previous studies have identified novel lncRNAs across specific cell types, to our knowledge, none have performed a cross species analysis of their lncRNAs in canine lymphomas. A cross-species reference in dog lymphomas additionally strengthens our claim for the existence of lymphoma specific lncRNAs and may facilitate focused anti-lncRNA therapeutic trials in dogs.

Finally, a number of supporting evidences point to a potential driver role for at least some of our novel lncRNAs. First we find that several lncRNAs are highly expressed in only a few tumor samples, reminiscent of aberrant over-expression due to gene fusions. We discovered many novel mutations in the exonic regions of these novel lncRNAs and subsequent analysis suggests that these mutations may frequently stabilize the lncRNAs secondary structures; this suggests frequent gain of regulatory function due to increased lncRNA stability, perhaps reinforcing epigenetic deregulation linked with lymphomagenesis [45].

## Conclusions

This exhaustive analysis of novel lncRNAs in DLBCL using RNA-seq from primary tumors, uncovers novel lncRNAs with our validations suggesting their potential regulatory roles and functions in DLBCL tumorgenesis. The cross-species analysis cross-validates the existence of these lymphoma specific lncRNAs and could potentially serve as a basis for future lymphoma clinical trials in Dogs. In addition to expanding the DLBCL transcriptome, these lncRNAs also provide abundant source of new targets for antisense oligonucleotide pharmacology.

## Additional files

Additional file 1:
**List of cell lines.** Data include list of DLBCL cell line names used in the analysis. (XLSX 38 kb) Additional file 2:
**Genomic coordinates of lncRNAs.** Data include genomic coordinates of the 2,632 lncRNAs with start, end coord, and IDs. Also consists of multiple sheets with additional information on mutation hotspots, genomic coordinates, and IDs for the 45 highly expressed lncRNAs and the divergent lncRNAs. (XLSX 896 kb) Additional file 3: 
**Figure S1**. lncRNA overlap with other Cancers. (PDF 259 kb) Additional file 4:
**Figure S2**.  Differential expression lncRNAs across Naïve B cells and Centroblasts. (PDF 425 kb) Additional file 5:
**Figure S3**. Differential expression of lncRNAs across Normal B Cells and Primary Tumors (PDF 1368 kb) Additional file 6:
**Figure S4**. Divergently Transcribled lncRNAs. (PDF 288 kb) Additional file 7:
**List of protein-coding genes correlated with lncRNAs.** Each protein-coding gene co-expressed with a novel lncRNA, with spearman correlation value, *P* value, and adjusted *P* value, respectively. The list includes most significantly positively correlated genes per lncRNA. (XLSX 23304 kb) Additional file 8:
**lncRNAs correlated with protein-coding genes and functional pathways.** List of lncRNAs significantly co-expressed with protein-coding genes and corresponding enriched functional pathways based on the subset of the co-expressed genes. (XLS 286 kb) Additional file 9:
**DLBCL sample subtype classification.** Data showing the classification of the 104 primary DLBCL subtypes as ABC or GCB. (XLSX 37 kb) Additional file 10:
**Figure S5**. Differentially expressed known lncRNAs across ABC and GCB subtypes. (PDF 1956 kb) Additional file 11:
**Figure S6**. Visualizing novel lncRNAs in reference to super enhancers. (PDF 404 kb) Additional file 12:
**Figure S7**. TSS plot for protein coding genes. (PDF 337 kb) Additional file 13:
**Figure S8**. Transposable element overlap for Known lncRNA transcripts. (PDF 238 kb) Additional file 14:
**lncRNAs overlapping and correlated with known datasets.** Data showing results for each lncRNA analyzed. Information included for each lncRNA, showing number of isoforms, lncRNA present in cell lines, normal B cell samples, near enriched ChIPSeq peaks, DLBCL super enhancers, PacBio data. Also, lncRNAs significantly co-expressed with known DLBCL target genes such as EZH2, BCL6. (XLSX 170 kb)

## Abbreviations

FDR: False Discovery Rate
FPKM: Fragments per kilobase of transcript per million mapped reads
GTF: Gene Transfer Format
lncRNA: Long Non-Coding RNA
SNV: Single Nucleotide Variant
TE: Transposable Elements
