# Supplementary figures and images for: Transcriptome sequencing reveals thousands of novel long non-coding RNAs in B cell lymphoma
Source: Genome Med. 2015 Nov 1;7:110. doi: 10.1186/s13073-015-0230-7 (PMC4628784; doi:10.1186/s13073-015-0230-7)

Fig S1

**Percentage of Novel IncRNA Overlapping  
with other Cancer IncRNA**

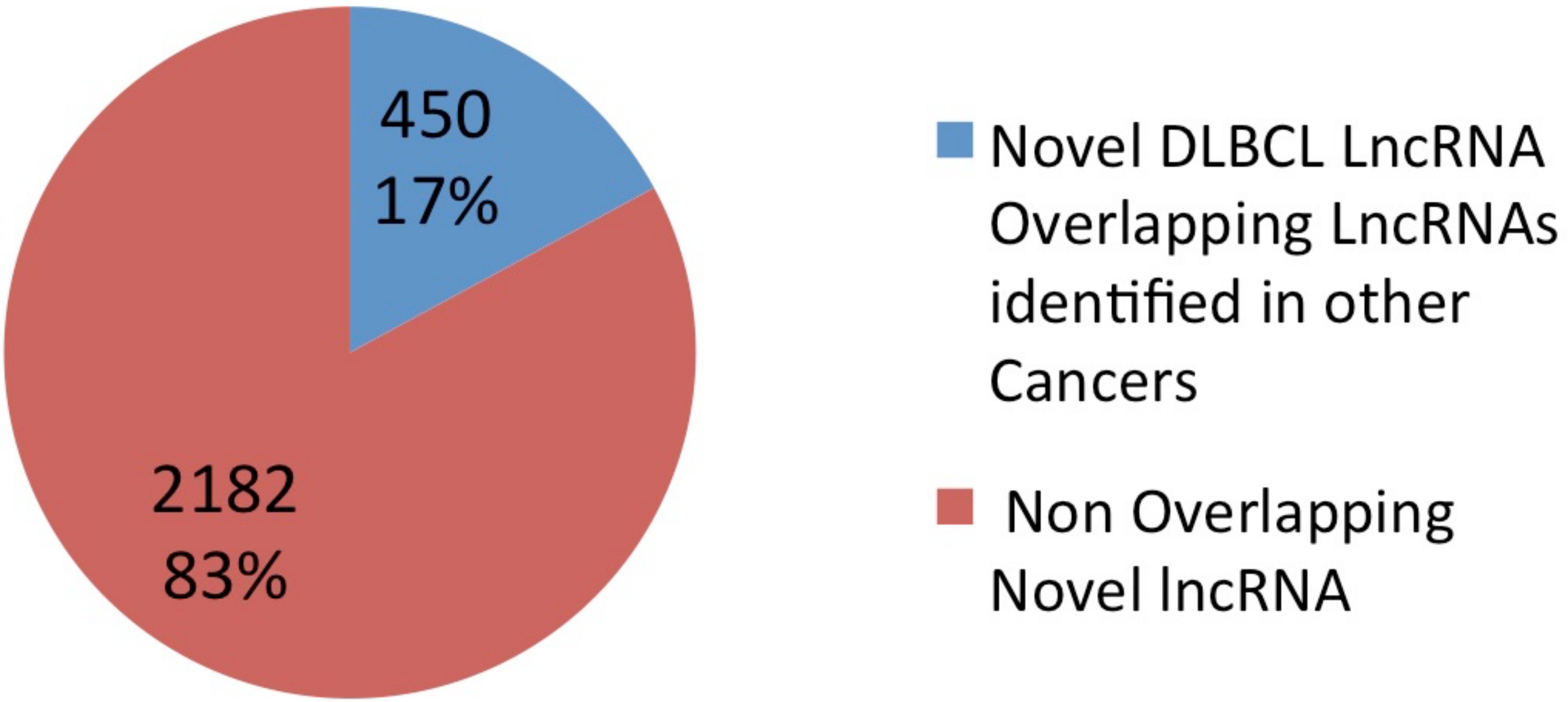

Supplement: Additional file 3: — Figure S1. lncRNA overlap with other Cancers. (PDF 259 kb) [file 13073_2015_230_MOESM3_ESM.pdf]

Fig S4

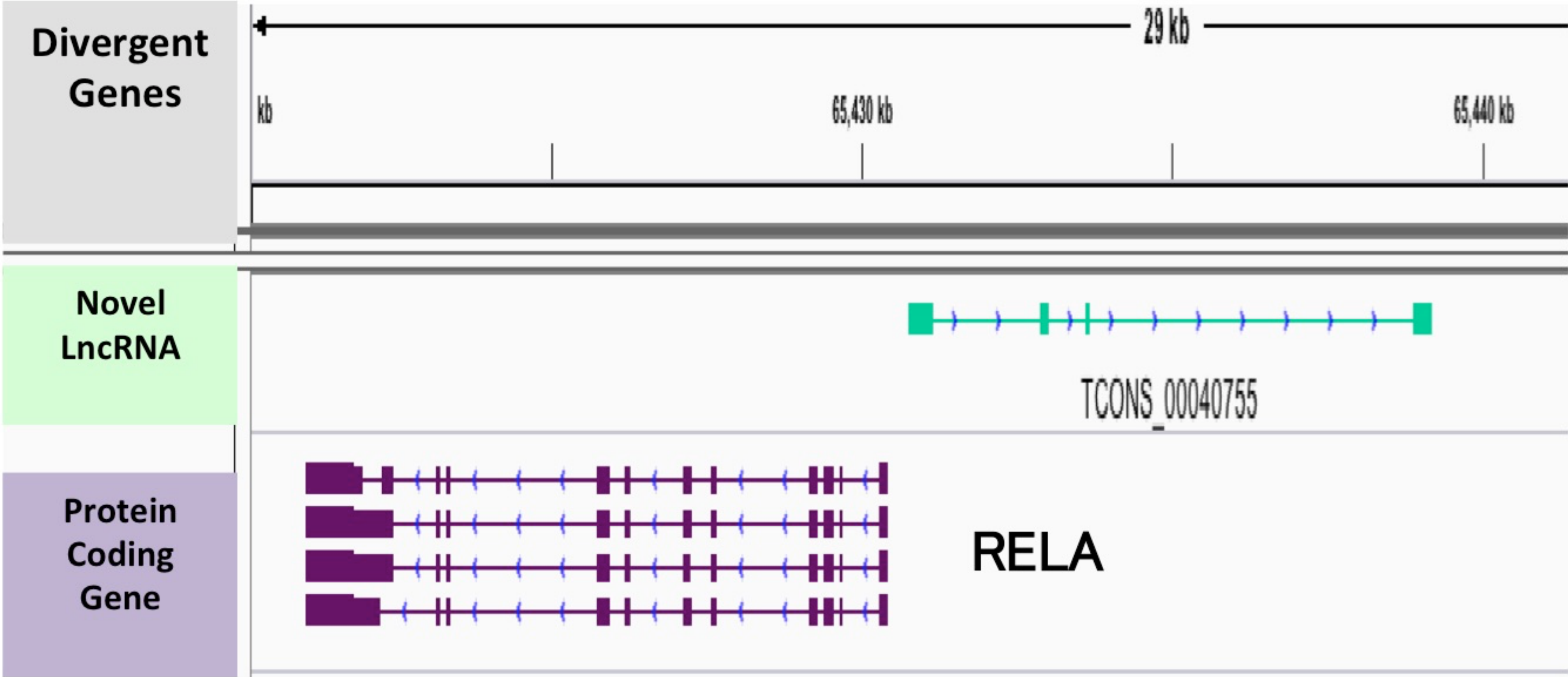

Supplement: Additional file 6: — Figure S4. Divergently Transcribled lncRNAs. (PDF 288 kb) [file 13073_2015_230_MOESM6_ESM.pdf]

Fig S6

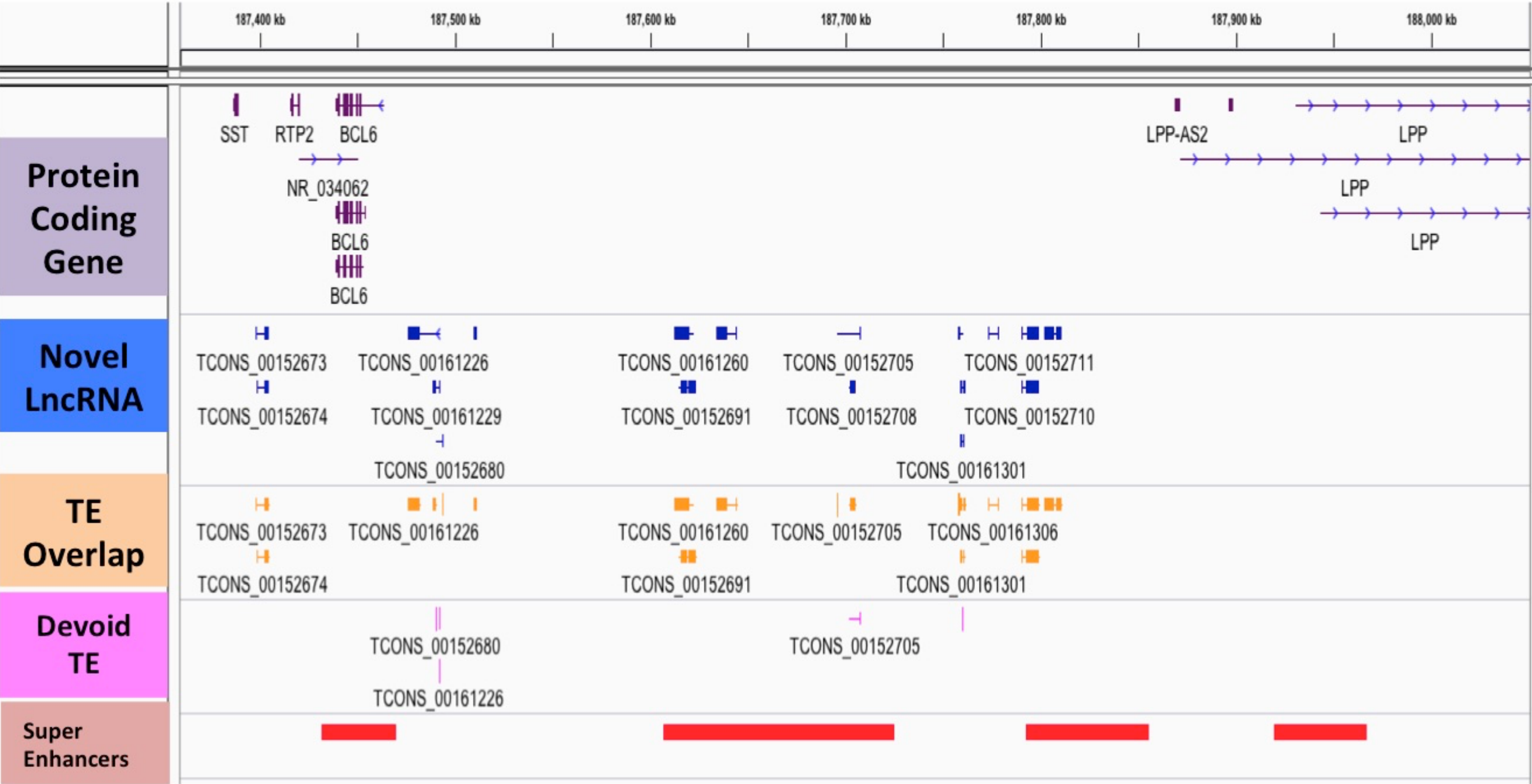

Novel LncRNAs and Super Enhancers near BCL6

Supplement: Additional file 11: — Figure S6. Visualizing novel lncRNAs in reference to super enhancers. (PDF 404 kb) [file 13073_2015_230_MOESM11_ESM.pdf]

Fig S8

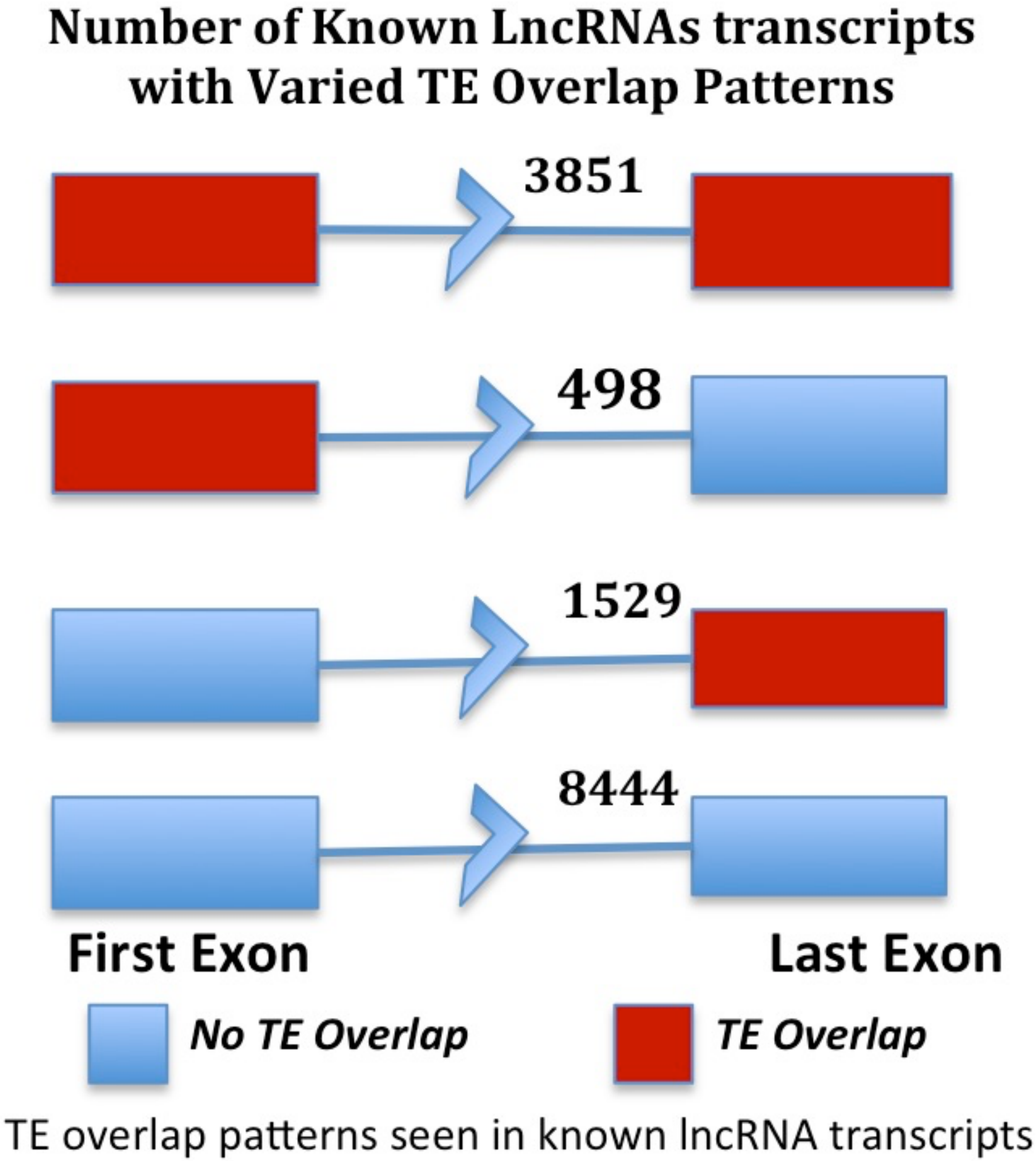

Supplement: Additional file 13: — Figure S8. Transposable element overlap for Known lncRNA transcripts. (PDF 238 kb) [file 13073_2015_230_MOESM13_ESM.pdf]
