# Supplementary material for: Transcriptome sequencing reveals thousands of novel long non-coding RNAs in B cell lymphoma
Source: Genome Med. 2015 Nov 1;7:110. doi: 10.1186/s13073-015-0230-7 (PMC4628784; doi:10.1186/s13073-015-0230-7)
Supplement: Additional file 4: — Figure S2. Differential expression lncRNAs across Naïve B cells and Centroblasts. (PDF 425 kb) [file 13073_2015_230_MOESM4_ESM.pdf]

Fig S2

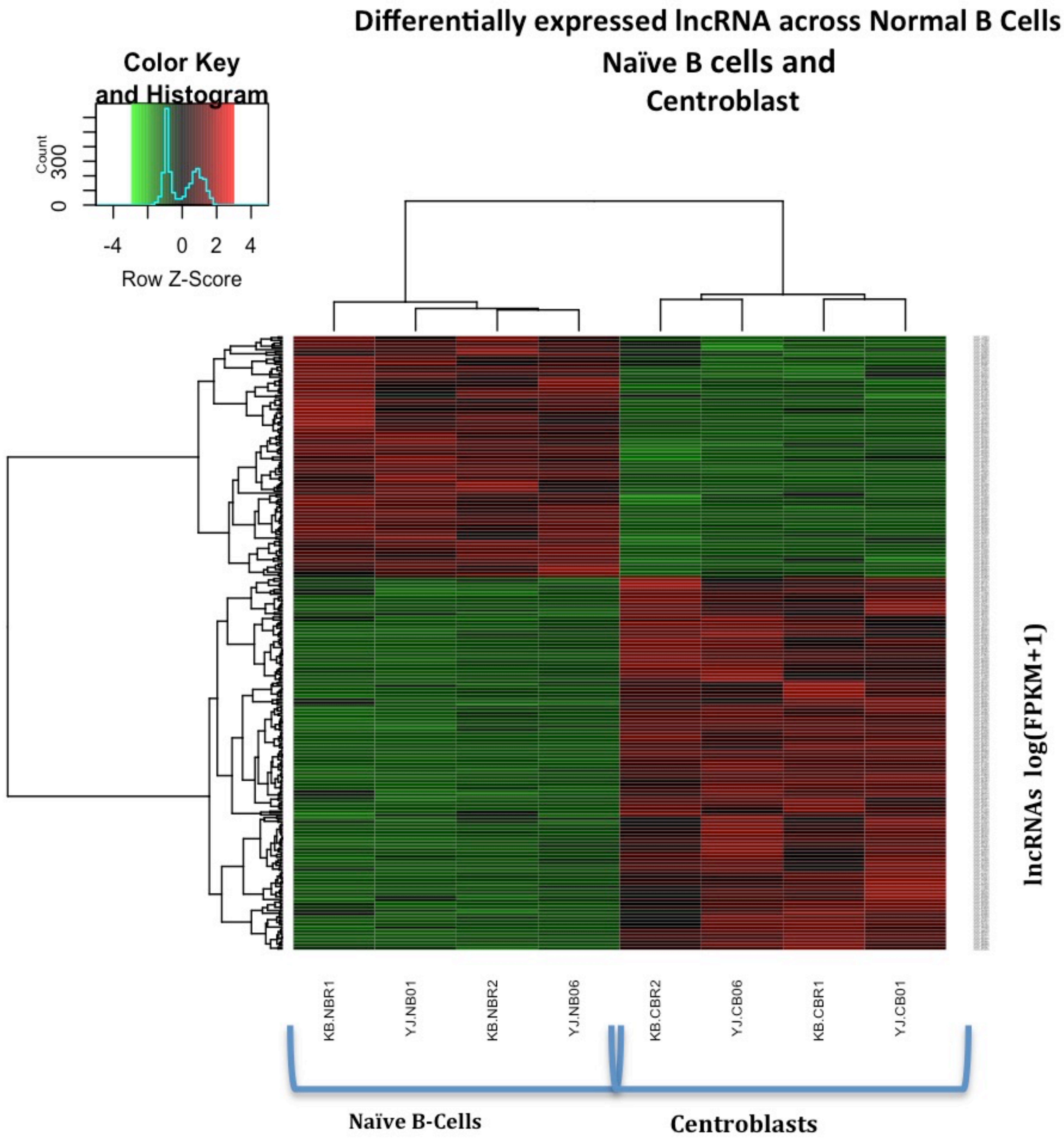

Differential expression between Normal B cells Subtypes – Naïve B cells and GCB, show 334 Novel lncRNAs significantly differentially expressed (FDR) < 0.05
