# Supplementary material for: Transcriptome sequencing reveals thousands of novel long non-coding RNAs in B cell lymphoma
Source: Genome Med. 2015 Nov 1;7:110. doi: 10.1186/s13073-015-0230-7 (PMC4628784; doi:10.1186/s13073-015-0230-7)
Supplement: Additional file 5: — Figure S3. Differential expression of lncRNAs across Normal B Cells and Primary Tumors (PDF 1368 kb) [file 13073_2015_230_MOESM5_ESM.pdf]

Fig S3

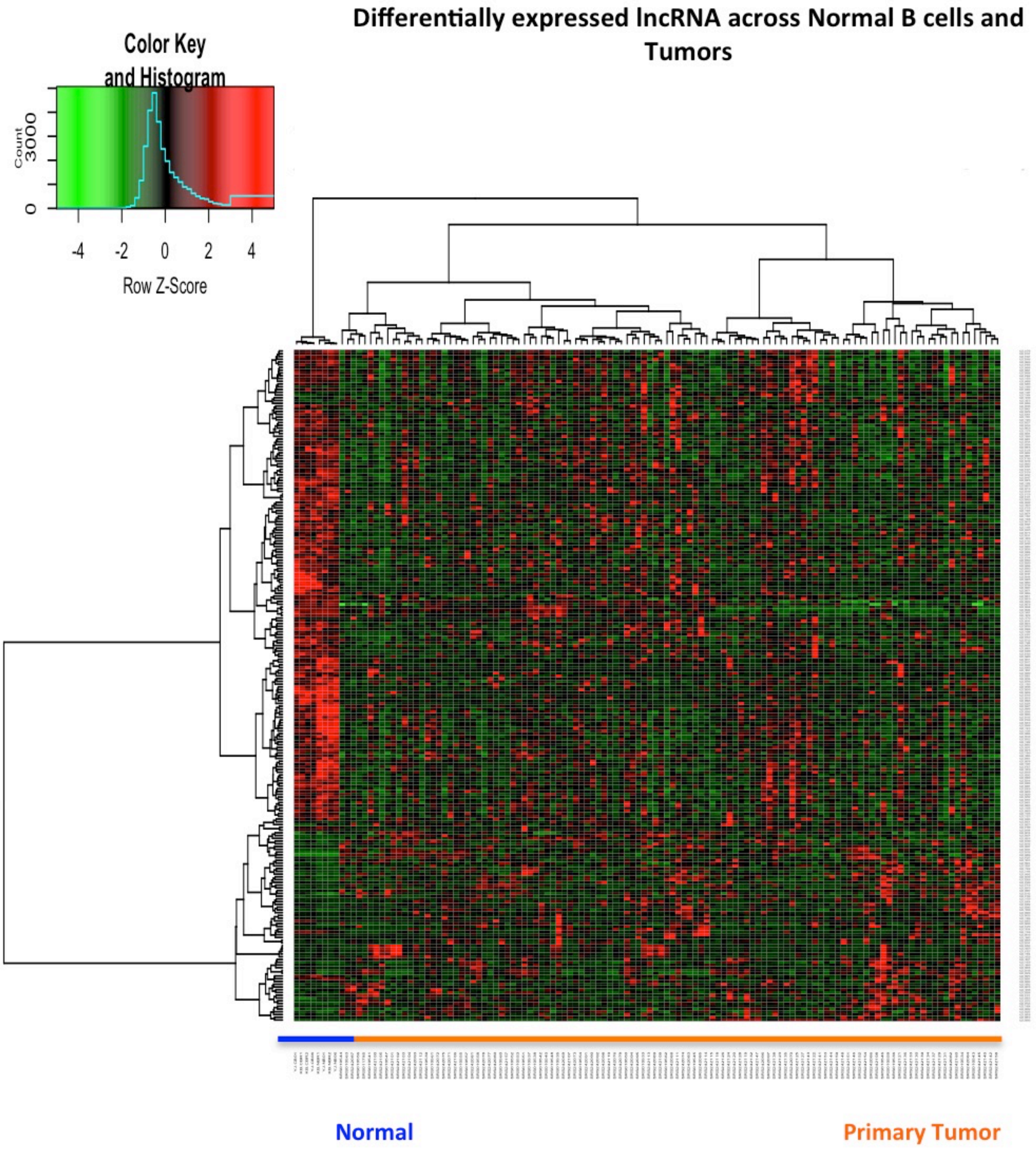

Differential expression analysis between Normal B cells (Naïve and GCB), show 1090 Novel lncRNAs significantly differentially expressed (FDR) < 0.05
