# Supplementary material for: Transcriptome sequencing reveals thousands of novel long non-coding RNAs in B cell lymphoma
Source: Genome Med. 2015 Nov 1;7:110. doi: 10.1186/s13073-015-0230-7 (PMC4628784; doi:10.1186/s13073-015-0230-7)
Supplement: Additional file 10: — Figure S5. Differentially expressed known lncRNAs across ABC and GCB subtypes. (PDF 1956 kb) [file 13073_2015_230_MOESM10_ESM.pdf]

Fig S5

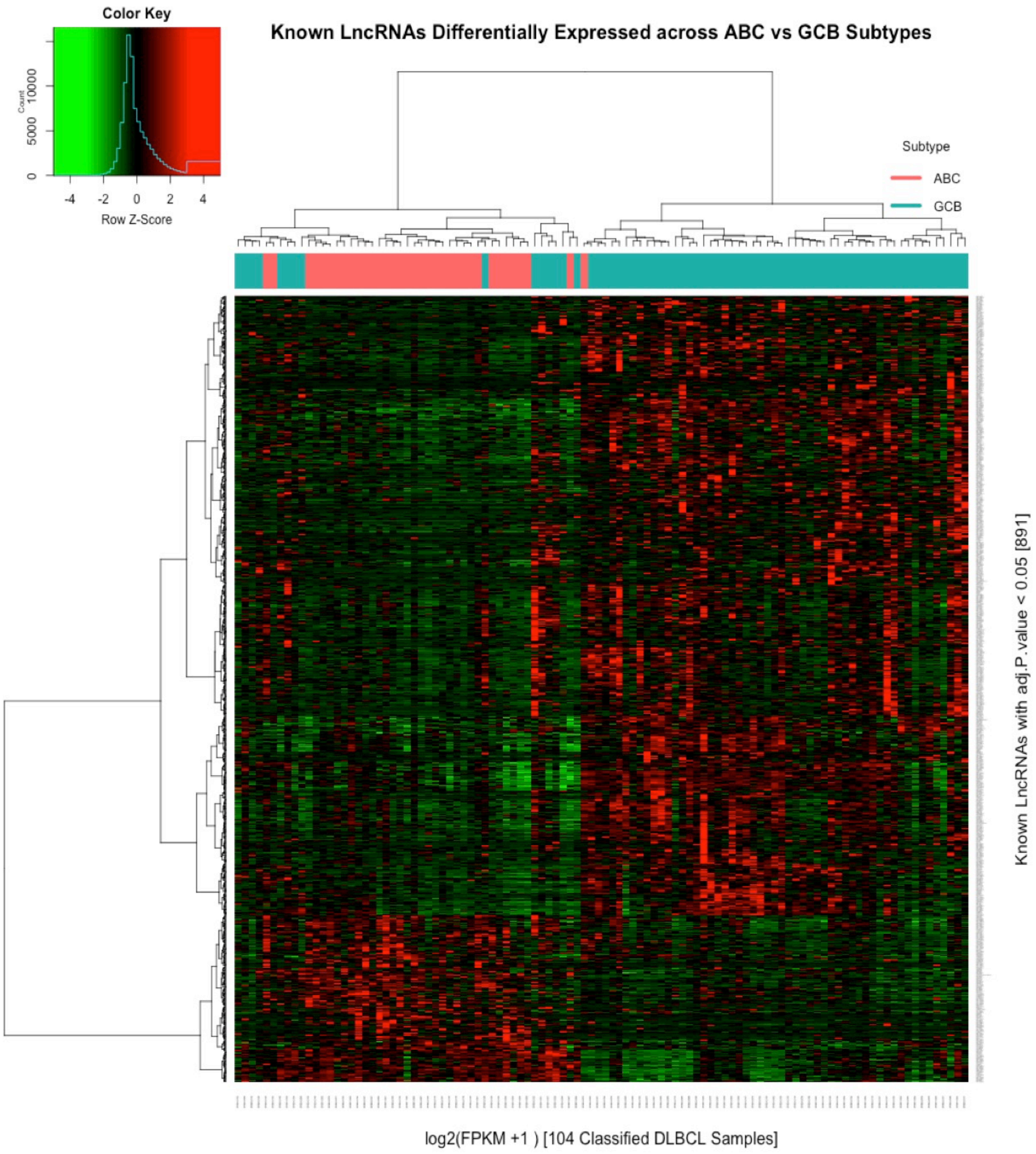

Differential expression of Known LncRNAs between ABC and GCB subtypes show 678 Novel lncRNAs significantly differentially expressed (FDR) < 0.05
