# Supplementary material for: Transcriptome sequencing reveals thousands of novel long non-coding RNAs in B cell lymphoma
Source: Genome Med. 2015 Nov 1;7:110. doi: 10.1186/s13073-015-0230-7 (PMC4628784; doi:10.1186/s13073-015-0230-7)
Supplement: Additional file 12: — Figure S7. TSS plot for protein coding genes. (PDF 337 kb) [file 13073_2015_230_MOESM12_ESM.pdf]

Fig S7

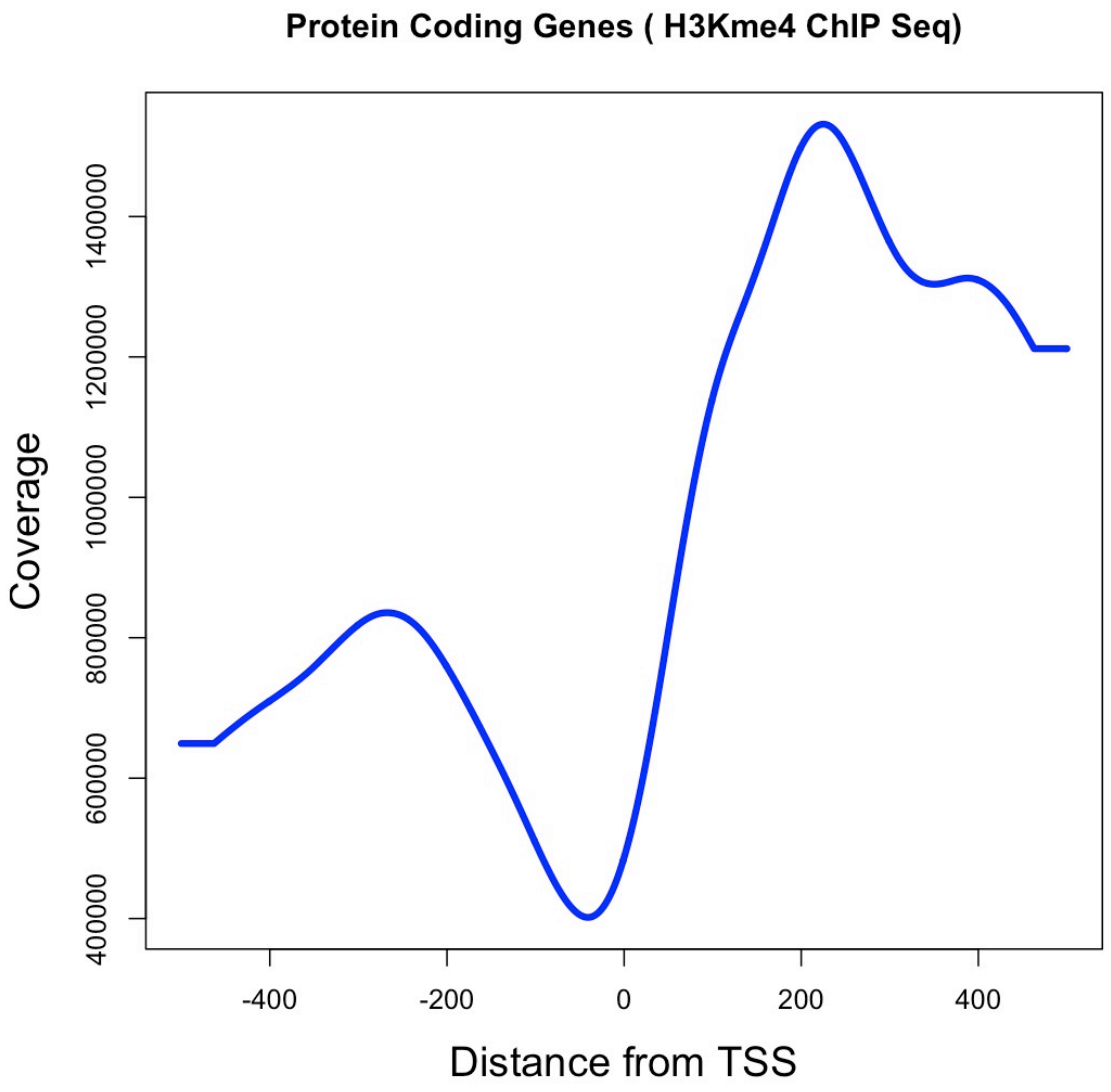

Transcription Start Site plot showing H3K4me3 read coverage across the entire genome at and around the TSS of Protein coding genes (RefSeq), with a nucleosome free region slightly upstream of the TSS and a +1 nucleosome downstream
